# Size-Dependent Biological Effects of Quercetin Nanocrystals

**DOI:** 10.3390/molecules24071438

**Published:** 2019-04-11

**Authors:** Qian Liu, Xi Yang, Jianxu Sun, Fanglin Yu, Hui Zhang, Jing Gao, Aiping Zheng

**Affiliations:** State Key Laboratory of Toxicology and Medical Countermeasures, Beijing Institute of Pharmacology and Toxicology, 27th Taiping Road, Haidian District, Beijing 100850, China; liu__qian1991@163.com (Q.L.); zzwyx1119@126.com (X.Y.); sunjianxu409@126.com (J.S.); yufanglin79@163.com (F.Y.)

**Keywords:** quercetin, nanocrystals, anti-tumor, invasion, signal transducer and activator of transcription 3 signal pathway

## Abstract

Quercetin (QE) is an attractive natural compound for cancer prevention due to its beneficial anti-oxidative and anti-proliferative effects. However, QE is poorly soluble in water and slightly soluble in oil, which results in its low oral bioavailability and limits its application in the clinic. The aim of this study was to prepare QE nanocrystals (QE-NCs) with improved solubility and high drug loading, furthermore, the size-dependent anti-cancer effects of QE-NCs were studied. We prepared QE-NCs with three different particle sizes by wet milling, then, cell proliferation, migration and invasion were studied in A549 cells. The QE-NCs had antitumor effects in a dose- and size-dependent manner. Compared with the large particles, the small particles had a strong inhibitory impact on cell biological effects (*p* < 0.05 or *p* < 0.01). Moreover, Western blot assay indicated that QE-NCs may inhibit the migration and invasion of A549 cells by inhibiting the STAT3 signaling pathway, and the particle size may have an effect on this process. In this study, it was proven that NCs could dramatically enhance the anticancer efficacy of QE at the cellular level. In addition, particle size had a considerable influence on the dissolution behavior and antitumor effects of NCs.

## 1. Introduction

Quercetin (QE) and related flavonoids are widely distributed in plants and have exhibited high activity toward anti-oxidative, anti-inflammatory, anti-microbial [1]. Besides, recent studies have found that QE can restrain the proliferation and metastasis of multiple cancer cell types, such as prostate cancer [2], breast cancer [3], colon cancer [4], lung cancer [5] and pancreatic cancer cells [6]. Document results revealed the mechanism may be associated with the regulation of various signaling pathways. QE could induce apoptosis in human lung adenocarcinoma cell line A549 through mitochondrial depolarization by down-regulating the interleukine-6/signal transducer and activator of transcription 3 (IL-6/STAT3) signaling pathway [7], and QE could also inhibit the proliferation of cancer cells by regulating the phosphoinositide 3-kinase (PI3K), mitogen-activated protein kinase/extracellular signal-regulated kinase (MEK/ERK) and epidermal growth factor receptor (EGFR) signaling pathway [8,9,10]. Moreover, QE has demonstrated advantages in reducing the multidrug resistance (MDR) of tumor cells. QE could increase intracellular accumulation of doxorubicin in breast cancer cells through down-regulating the expression of efflux ABC transporters, which can effectively potentiate the anti-tumor effect of doxorubicin at a lower concentration and attenuate the toxic side effects of it [11]. The combination of QE and chemotherapy drugs could improve the overall anti-cancer efficacy.

However, QE is poorly soluble in water and is slightly solubilized in most pharmaceutically acceptable solvents, resulting in its low bioavailability [12,13]. It was reported that the bioavailability of QE was less than 10%, which limited its clinical application [13]. In order to overcome these disadvantages, some modified dosage forms of QE have been developed, mainly including nanoparticles [14], microemulsions [15] and other drug carriers [16,17]. Nanoparticles need a large amount of carrier materials and had a low drug loading. Microemulsions required a large number of surfactants and had potential side effects. Therefore, there is a need for developing convenient and appropriate methods to produce the desired QE formulations.

A nanocrystal (NC) suspension is a colloidal system that consists of only pure drug crystals and minimum surfactants for stabilization [18]. The formulation can present a drug loading as high as 100% and increase the solubility and dissolution of poorly water-soluble drugs. It requires few chemical solvents and thus reduces excipient toxicity. There have been several studies to develop such formulations [19,20]. Sahoo [21] fabricated QE-NCs using high-pressure homogenization with an average particle size of 483 nm, and the solubility of QE-NCs was about 20-fold that of crude QE. Sun [22] prepared QE-NCs by a tandem of nano-precipitation (NP) and high-pressure homogenization (HPH) method with an average particle size of 393.5 nm. The solubility of QE-NCs was about 70-fold that of crude QE. Compared with the control group, the C_max_ of QE-NCs increased by 2 times, and the relative bioavailability was 15 times higher, which significantly increased the solubility and the oral absorption of QE. However, most of the present researches focused on the preparation and pharmacokinetics of QE-NCs, and the effect on the anti-tumor activities of QE-NCs has rarely been investigated. It is worth exploring whether the increase of solubility of QE-NC will affect its biological effects, and how particle size affects this process.

With a view of that QE-NC formulations need further optimization and the study of QE-NCs anticancer effects remains incomplete, we prepared QE-NCs with three markedly different particle sizes by wet milling; then, cell proliferation, adhesion, migration, and invasion were studied in A549 cells. The main purposes of this study include exploring the anticancer effects of QE-NCs, and investigating whether the particle size of QE-NCs affects their biological effects.

## 2. Results

### 2.1. Preparation and Characterization of QE-NCs

**Figure 1:**
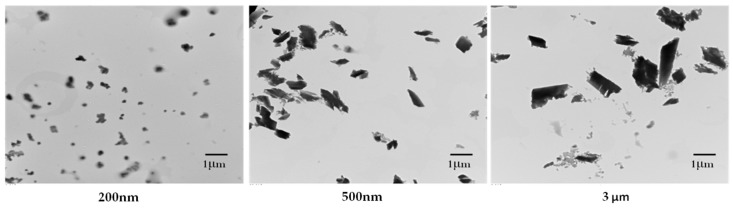
Characterization of QE-NCs with different sizes from transmission electron microscopy (TEM) observation.

QE-NCs of different sizes were prepared by a wet milling method, and the morphological features of the QE-NCs were characterized by transmission electron microscopy (TEM). The mean particle size, polydispersity index (PDI) and zeta potential of the QE-NCs are shown in Table 1. The mean particle sizes of the three prepared QE-NCs were approximately 200 nm, 500 nm, and 3 μm. The results demonstrate that the QE-NCs showed a narrow size distribution and better physical stability. TEM images revealed that the three different sizes of NCs were uniformly distributed with a cone-shaped morphology (Figure 1).

### 2.2. Effect of QE-NCs on Cell Proliferation

**Figure 2:**
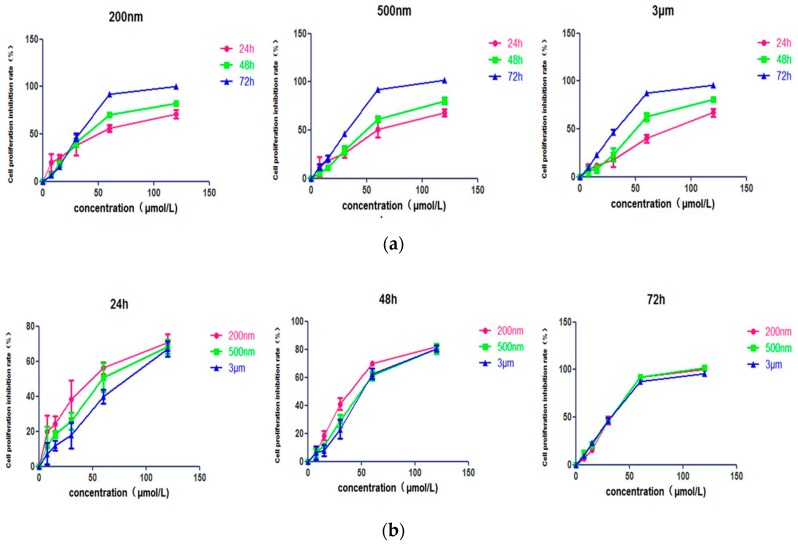
Characterization of cell proliferation. (**a**) Effect of different concentrations of QE-NCs on the proliferation of A549 cells. (**b**) Effect of different particle sizes of QE-NCs on the proliferation of A549 cells (*n* = 6).

A549 cells were subjected to the cell counting kit-8 (CCK-8) assay to evaluate the anticancer effect of QE-NCs and the effect of different particle sizes on cell proliferation. First, QE-NCs reduced the proliferation of A549 cells in a concentration- and time-dependent manner (Figure 2a). The results showed that with the increase of drug concentration and prolongation of treatment time, the inhibition effects of QE on A549 cells were enhanced, indicating that QE may have latent clinical applicative value. Second, the particle size of QE-NCs had a significant effect on the proliferation of A549 cells. The results demonstrate that the 200 nm and 500 nm particles showed higher cell proliferation resistance than did the 3 μm particles at the six concentrations (0, 7.5, 15, 30, 60, and 120 μmol/L) studied (Figure 2b), and the smaller the particle size, the more obvious the effect. Third, the half maximal inhibitory concentrations (IC_50_) values at 24, 48, and 72 h are shown in Table 2. After a 24 h culture period, the IC_50_ values of 200 nm, 500 nm and 3 μm particles were (47.03 ± 16.64), (62.25 ± 15.65) and (77.06 ± 12.29) μmol/L, respectively. The IC_50_ value of the 3 μm group was 1.63 times higher than that of the 200 nm group, the advantage of the smaller particle size anti-cell proliferation effects is obvious. There was no difference in IC_50_ between the three particle size groups after a 72 h culture period. The results revealed that particle size can inhibit the proliferation of A549 cells in terms of speed and extent, and the smaller the particle size, the stronger the effect.

### 2.3. Effect of QE-NCs on Cell Adhesion

**Figure 3:**
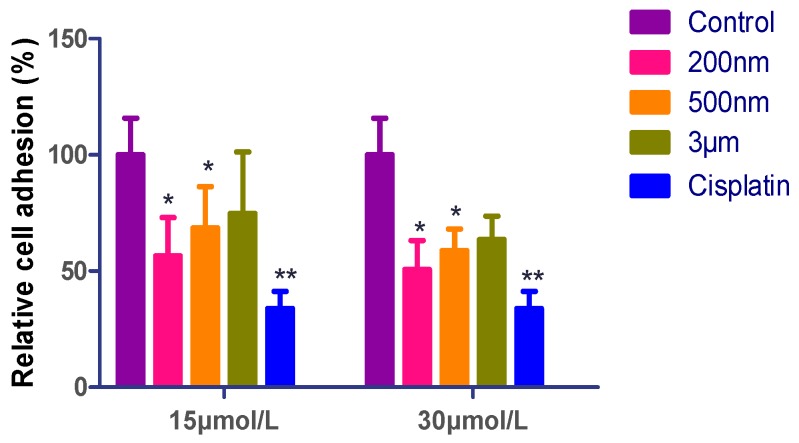
Effects of different sizes and concentrations of QE-NCs on the adhesion rate of A549 cells (*n* = 6). * *p* < 0.05, ** *p* < 0.01, compared with the blank control group.

To explore the effect of QE-NCs on the adhesion of A549 cells, a matrix adhesion assay was conducted to test the adhesion of A549 cells following treatment with QE-NCs for 24 h. As indicated in Figure 3, the result showed the effect of the concentrations of QE-NCs on cell adhesion. Compared with 15 μmol/L, the adhesion was subjected to greater damage or poorer formation at 30 μmol/L (*p* < 0.05), showing a dose-dependent pattern. In addition, at the concentration of 30 μmol/L, the average adhesion rate of cells when treated with 500 nm QE-NCs decreased to 58.79% of control levels (*p* < 0.05) and reduced further to 50.76% of control levels after being treated with 200 nm QE-NCs (*p* < 0.05). The adhesion rate of the 3 μm group was decreased, but there was no significant difference compared with the control group (Table 3). The particle size of QE-NCs has a significant effect on cell adhesion. These results show that smaller particle sizes and higher concentrations resulted in greater damage or poorer formation of the microfilaments; the small particles might sterically block the normal localization of the actin fibers and cause the disruption and remodeling of the actin cytoskeleton [23].

### 2.4. Effect of QE-NCs on Cell Migration

**Figure 4:**
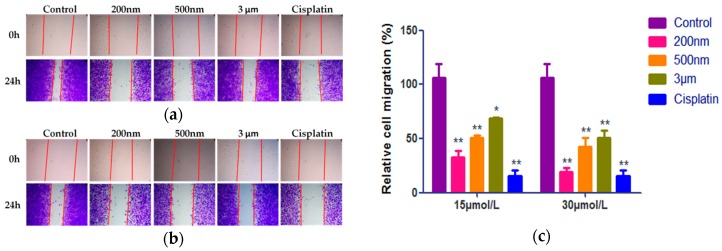
Effects of different sizes and concentrations of QE-NCs on the mobility rate of A549 cells (*n* = 3). (**a**) Results of the wound healing assay with treated with 15 μmol/L QE-NCs. (**b**) Results of the wound healing assay with treated with 30 μmol/L QE-NCs. (**c**) Statistical analysis results. * *p* < 0.05, ** *p* < 0.01, compared with the blank control group.

To probe the effect of QE-NCs on the metastatic capability of A549 cells, the wound healing assay was carried out. The wound healing assay involved the application of a wound to a monolayer of cells and subsequently measuring the closed distance over time as compared to a control [24]. There was a positive correlation between the speed/degree of scratch healing and the ability of cell migration, which mimicked the migration of cells in vivo to some extent [25,26]. It can be seen visually from the picture of the scratch repair experiment (Figure 4a,b). After 24 h of culture, many cells treated with 3 μm QE-NCs migrated to the center of the scratch field, while few cells treated with 500 nm QE-NCs and even fewer cells treated with 200 nm QE-NCs did so. Pictures were analyzed using ImageJ 1.46r analysis software (National Institutes of Health, Bethesda, MD, USA), and data are presented as the percentage of cell migration compared to the blank control (Figure 4c, Table 4). The results pointed out that the QE-NCs of three particle sizes had significant differences compared to the control group, indicating that QE could inhibit the migration of A549 cells. As the increasing of the concentration, the ability of migration inhibition increased, while the difference between the high-concentration group of 200 nm QE-NCs and the positive control group was not statistically significant. Besides, the mobility inhibition rate of A549 cells increased with the decreasing particle size of QE-NCs, the effects of the 200 nm particle size groups were significantly different from the 500 nm and 3 μm particle size groups (*p* < 0.01 or *p* < 0.05).

### 2.5. Effect of QE-NCs on Cell Invasion

**Figure 5:**
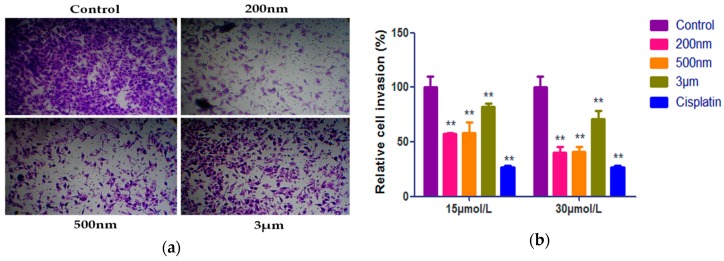
Effects of different sizes and concentrations of QE-NCs on the invasion ability of A549 cells (*n* = 3). (**a**) Results of the transwell chamber invasion assay. (**b**) Statistical analysis of the results of the transwell chamber invasion assay. ** *p* < 0.01, compared with the blank control group.

To further investigate the effect of QE-NCs on the cell migration ability of A549 cells, a Matrigel invasion assay was evaluated using transwell chambers. Figure 5a shows representative pictures for cells treated with QE-NCs and the blank control group. The results consistent with the cell migration assay can be seen intuitively from the figure, namely, the cell invasion inhibitory effects of nano-particle size were stronger than that of micron particle size. Moreover, a quantitative analysis illustrated that treatment with QE-NCs considerably inhibited the invasion of A549 cells (Table 5). The cell invasion ability of the three particle size QE-NCs in each concentration group decreased significantly with the increase of the concentration. The difference was statistically significant compared with the blank control group (*p* < 0.01), and the high concentration groups of 200 nm and 500 nm particle size have no significant difference compared with the positive control cisplatin group. The particle size also has a significant effect on cell invasion. The relative invasion of A549 cells treated with 200 nm QE-NCs was extensively decreased (*p* < 0.05) compared with that of A549 cells treated with 500 nm QE-NCs, and yet lower (*p* < 0.01) than that of cells treated with 3 μm QE-NCs. The results show that particle size strongly affects the invasion of A549 cells, and they are consistent with those of the cell migration assay.

### 2.6. Effect of QE-NCs on STAT3 Expression

**Figure 6:**
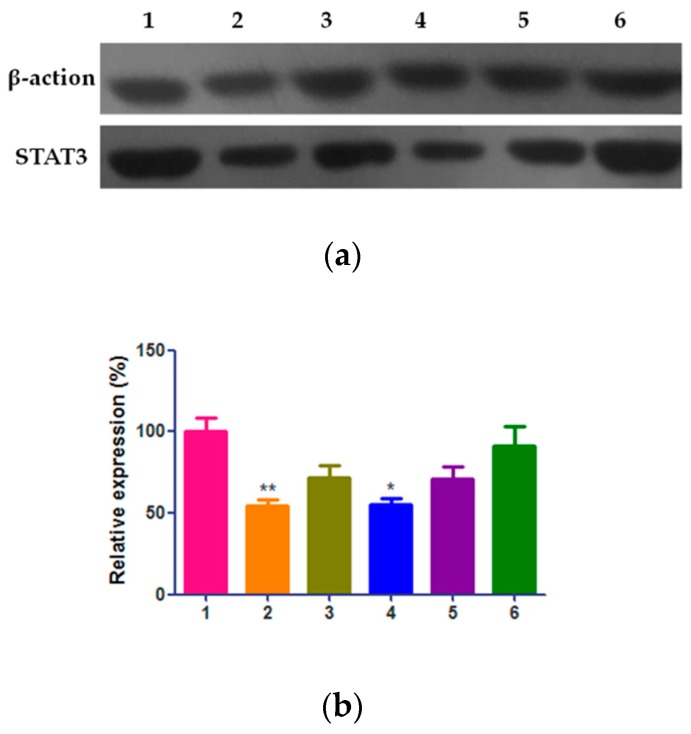
Effect of QE-NCs with different sizes on the protein level of STAT3 in A549 cells (*n* = 3). (**a**) Western blot results. (**b**) The results of statistical analysis. 1. Blank control group; 2. Cisplatin 3 μg/mL group; 3. QE-NCs (200 nm, 15 μmol/L) group; 4. QE-NCs (200 nm, 30 μmol/L) group; 5. QE-NCs (500 nm, 30 μmol/L) group; 6. QE-NCs (3 μm, 30 μmol/L) group. * *p* < 0.05, ** *p* < 0.01, compared with the blank control group.

Since the neoplastic phenotype of a cell is largely driven by aberrant gene expression patterns, increasing attention has been focused on transcription factors that regulate critical mediators of tumorigenesis, such as signal transducer and activator of transcription 3 (STAT3). To further discuss the exact molecular mechanism of biological effects induced by QE-NCs of different particle sizes, the expression levels of STAT3 were assessed. Our results from Western blotting showed that after treatment with QE-NCs for 24 h, STAT3 expression dramatically decreased (Figure 6a), and the smaller particle size and higher concentration resulted in higher efficiency. As the statistical results showed (Figure 6b), the expression of the protein in the 200 nm particle size group was significantly lower than that in the 500 nm and 3 μm group, and the difference was statistically significant (*p* < 0.05 or *p* < 0.01). The difference between the high concentration group of 200 nm particle size and the blank control group was statistically significant (*p* < 0.05), while the difference was not statistically significant compared with the positive control group. In summary, QE-NCs may inhibit the migration and invasion of A549 cells by inhibiting the STAT3 signaling pathway, and the particle size may have an effect on this process.

## 3. Discussion

QE is an attractive natural compound for potential cancer prevention due to its beneficial anti-oxidative, anti-proliferative, and anti-mutagenic capacity [27]. Its role in regulating cell signaling pathway, inhibiting cell metastasis and inducing apoptosis has been demonstrated in vitro and in animal studies [28,29,30]. Problems like poor aqueous solubility and low oral bioavailability, combined with the high dose required for treatment, make QE a defective candidate for therapeutic purposes [12,13]. Moreover, the rapid gastrointestinal metabolism of QE is also a major obstacle for its clinical translation [31]. Therefore, the development of modified formulations of QE with high drug loading, increased bioavailability, prolonged circulation time and decreased toxic effects at high doses is advocated. NCs have distinctive characteristics compared with other nanoformulations. NCs can not only present a drug loading as high as 100% but also reduce the toxic side effects caused by the excipients, resulting in satisfactory therapeutic concentrations at low doses [32]. Limited interactions between plasma proteins and NCs probably prevent therapeutic molecules from enzymatic metabolism, further increasing the circulation time and plasma concentrations of anticancer compounds [33,34]. NCs have shown promising results in the dissolution as well as the bioavailability of insoluble drugs.

Most of the previous research has studied the preparation and characterization of QE-NCs, and few studies systematically evaluated the anti-tumor effect of QE-NCs in vitro [21,35,36]. We prepared QE-NCs by wet milling. The minimum average particle size is about 200 nm, which is smaller than most of the previous studies. On this basis, the anti-tumor effect of QE-NCs was investigated from the molecular to the cellular level. We found that QE-NCs had negative effects on the cell proliferation, adhesion, migration, and invasion of A549 cells in a dose-dependent manner, indicating that QE-NCs may be a potential clinical medicine against cancer. The protein expression analysis suggested that QE-NCs reduced the expression of STAT3. In summary, QE-NCs can significantly restrain the proliferation, migration, and invasion of A549 cells and its mechanism is probably related to the inhibition of the STAT3 signaling pathway.

Compared with other nanoformulations, QE-NCs also have some advantages in anti-tumor effects. Baksi [37] developed QE loaded chitosan nanoparticles (QE-CS-NPs) with an encapsulation efficiency of 79.78%, and the IC_50_ values of QE-CS-NPs on A549 cells for 48 h was about 80 μmol/L. Lakshmi [38] prepared QE-mediated gold nanoclusters (QE-GNCs) and the IC_50_ values of QE-GNCs on A549 cells for 24 h were about 300 μmol/L. Tan [16] prepared QE-loaded nanomicelles using the film casting method. The incorporation efficiency into the nanomicelles was ≥88.9% and the IC_50_ value of the nanomicelles on A549 cells for 72 h was about 200 μmol/L. Corresponding to above nanoformulations, the IC_50_ values at 24, 48, and 72 h of QE-NC with 200 nm particle size were 47, 38 and 30 μmol/L. QE-NCs could improve the solubility of insoluble drugs, with almost unrestricted entrapment efficiency/drug loading, which demonstrated a higher anticancer activity and resulted in therapeutic effect at low doses.

Moreover, to further understand the biological effects of NCs and then optimize the formulation, investigating the complicated molecular aspects of particle size–bio-interactions is meaningful. In particular, previous research has been focused on particle sizes smaller than 200 nm, while NCs possess a mean crystal size between 200 and 800 nm [39]. Our results found that QE-NCs had effects on cell proliferation, adhesion, and migration in a size-dependent manner. The expression of STAT3 protein reduced as the particle size decreased.

There are some possible explanations for these results. QE-NCs can affect the cell biological effects in two ways—first, by influencing protein adsorption around the cells, and second, by being uptaken directly by cells; the degree of this in both cases largely depend on nanotopography and particle size [23]. When NCs enter the physiological environment, the surface of NCs may adsorb proteins to form protein crowns. This kind of NCs-protein complexes can directly affect the various reactions of NCs, such as cell uptake, signal transduction, biological distribution and toxicity [40,41,42], and the smaller the particle size, the more obvious the effect. In addition, the specific surface can be increased as the size of NCs decreases, resulting in easier adsorption on the surface of tumor cells [42]. This interaction with the cell surface, on the one hand, can affect the normal localization of actin protein in space, and lead to the destruction and reconstruction of actin cytoskeleton, thus inhibiting the biological effects such as cell adhesion, migration, invasion [23]; on the other hand, NCs adsorbed on the cell surface can be ingested by cells through different ways. Particles of different sizes can enter cells through the following four ways: phagocytosis, macropinocytosis, and clathrin-mediated or caveolae-mediated endocytosis [43]. Particles with a size of 1 μm or greater are internalized via macropinocytosis, internalization of particles with a size smaller than 200 nm involves clathrin-coated pits, and with increasing size, the mechanism that relies on caveolae-mediated internalization becomes apparent; this becomes the main pathway of entry for particles of 500 nm size [44]. In our study, the sizes of QE-NCs were approximately 200 nm, 500 nm, and 3 μm. Therefore, the difference in the cellular internalization pathways of the three QE-NCs due to different sizes may be an important factor leading to the significant differences in antitumor effects.

Meanwhile, NCs are different from other nano-drug delivery systems in that NCs do not contain carriers, which NCs themselves interact with the biological system, resulting in a continuous dissolution process. As a direct result of decreased particle size, the solubility and dissolution rate of poorly water-soluble drugs can be dramatically enhanced. It is generally assumed that NCs dissolve faster than their microdimensional counterparts, leading to a high drug concentration gradient inside and outside the cell. Free drug molecules can diffuse passively through the biomembranes and reduce their pharmacodynamic action [32]. The exact contribution of each factor needs to be investigated in greater depth. Further, some studies have shown that the effect of particle size on cell endocytosis is related to the chemical composition of the particles and the type of cells. Because of the membrane structure of various cells and the presence of different proteins, different cell types can have an effect on the total and kinetic processes of the endocytosed particles, resulting in differences in biological effects [45]. More research in this area will be done in the future.

Pure NCs are inclined to aggregation owing to their high surface area and high surface energy. Stabilizers such as surfactants are introduced in NC preparation to reduce the surface energy and yield stable NCs [46]. In the preparation of QE-NCs, stabilized NCs were formed by adding Tween 80 as a stabilizer, illustrating the excellent control of particle size that can be achieved with this method. Tween 80 adsorbed on the surface of NCs through physical interactions can not only control the particle size of QE-NCs but also prevent the agglomeration of QE-NCs. In addition, stabilizers can further endow NCs with additional functions such as prolonged circulation time [47], active tumor targeting [48], or anti-MDR [49]. Some studies have found that Tween 80 significantly reduced the expression of P-gp at non-cytotoxic doses [50]. Therefore, Tween 80 is likely to improve the exposure of P-gp substrates and enhance the local concentration of the drug in the tumor site, contributing to the improved therapeutic effect. This also suggests that the prepared QE-NCs have promising anticancer potential.

The advantages, such as ease to produce, high drug loading and physical stability, make QE-NCs attractive formulations for cancer resistance. In addition, QE-NCs can be applied to patients with a variety of administration routes, among which oral administration and intravenous injection are the most preferred [51]. QE-NCs can also be combined with chemotherapy drugs to enhance anti-tumor efficacy and reduce side effects [11,52,53]. Furthermore, the reduced toxicity of QE-NCs should be another advantage in clinical application [54], as some studies reported the toxic effects of QE at high doses in clinical trials [55]. QE-NCs have a higher anti-tumor activity and can reach effective therapeutic concentrations at low doses, which reduces the possibility of toxic effects. The QE-NCs can be further optimized by the addition of cancer cell-specific targeting moieties [1]. It will not only enhance target specific delivery of QE-NCs but will also reduce their interaction with the healthy cells, preventing the toxic effects.

It is worth noting that no new NC drugs have been approved since 2009 despite a few being tested in clinical trials [46]. One clear obstacle in commercializing NCs for delivering anticancer compounds is finding techniques that can produce stable and uniform NCs at the industrial scale. For this, it is the need of the hour to concentrate on NC preparation methods and performance. We believe that breakthroughs in developing novel NC methods and devices will occur in the near future.

## 4. Materials and Methods

### 4.1. Chemicals and Materials

Quercetin (purity > 98%) was purchased from Xi’an Changyue Biological Technology Co. Ltd. (Xi’an, China). CCK-8 chemical reagent was purchased from Bimake Company (Shanghai, China). BD Matrigel^TM^ Basement Membrane Matrix was purchased from BD Biosciences (San Jose, CA, USA). A transwell chamber was purchased from Corning (New York, NY, USA). STAT3 Mouse monoclonal antibody was received from Bimake Company (Shanghai, China). Dimethyl sulfoxide (DMSO) was purchased from Sinopharm Chemical Reagent Co. Ltd. (Shanghai, China). Methanol and acetonitrile (HPLC grade) are products of Thermo Fisher Scientific (Waltham, MA, USA). Horseradish-peroxidase-labeled goat anti-mouse IgG, an ECL Chemiluminescence kit, lysis buffer, and a BCA protein quantitative kit were purchased from Beijing DingGuoChangSheng Biological Technology Co. Ltd. (Beijing, China). Dulbecco’s modified Eagle’s medium (DMEM) and fetal bovine serum (FBS) were acquired from Gibco Technologies (Carlsbad, CA, USA).

### 4.2. Preparation and Characterization of QE-NCs

#### 4.2.1. Preparation of QE-NCs with Three Mean Particle Sizes

QE-NCs were prepared by a wet milling method. In brief, QE powder (10%, *w*/*v*) was dispersed in an aqueous surfactant solution containing 1% Tween 80 (*w*/*v*) under magnetic stirring. Then, the mixture was premilled by a scattered emulsification homogenizer-C25 (HENC, Shanghai, China) at 19,000 rpm for 5 min to obtain coarse drug suspensions. These coarse suspensions were further ground with zirconium oxide beads (0.3 mm diameter) in an AK71M-2WKF wet grinding machine (Willy A Bachofen AG, Muttenz, Switzerland). The three types of QE-NCs were prepared by simply changing the milling time and speed. Milling times and speeds are shown in Table 1.

#### 4.2.2. Characterization of QE-NCs

The particle size, PDI, and zeta potential of QE-NCs were measured by dynamic light scattering analysis (DLS) using a Malvern Zetasizer Nano ZS (Zetasizer Nano-ZS90, Malvern Instruments, Malvern, UK) at 25 °C. QE-NCs were diluted with deionized water at a ratio of 1:400 to a suitable scattering intensity before determination. The morphology of QE-NCs was determined using a transmission electron microscope (H-7650, Hitachi, Tokyo, Japan). QE-NCs were dropped onto a copper grid and then stained in phosphotungstic acid for the morphology study.

### 4.3. Cell Culture

Human lung adenocarcinoma cell line A549 was purchased from an American type culture collection (ATCC, Manassas, MD, USA). Cells were cultured in dulbecco’s modified eagle medium (DMEM) supplemented with 10% FBS and 1% penicillin/streptomycin at 37 °C in a humidified atmosphere containing 5% CO_2_. After achieving adherence for 24 h, cells were treated with or without QE-NCs at different concentrations. Cells cultured in DMEM alone were used as a negative control.

### 4.4. CCK-8 Proliferation Assay

The proliferation of A549 cells was examined using the CCK-8 assay kit. A549 cells were seeded in 96-well plates (Costar, New York, NY, USA) at a density of 3 × 10^3^ cells/well with 100 μL of DMEM for 12 h. After cell adhesion was achieved, the culture medium was replaced with 0, 7.5, 15, 30, 60, or 120 μmol/L QE-NCs with three different particle sizes. Then, the cells were incubated for another 24, 48, or 72 h. At specific time points, the plates were washed three times with PBS, and 100 μL of fresh DMEM without FBS and 10 μL of CCK-8 reagent were added. After 2 h of incubation, the absorbance of the supernatant at 450 nm was quantified using a microplate reader (Multiskan GO, Thermo Fisher Scientific, Waltham, MA, USA) to evaluate cell viability. The relative viability of the cells was calculated using the following formula: Cell proliferation inhibition rate (%) = (1 − As/Ac) × 100%, where As is the experimental optical density (OD) and Ac is the control OD. At least six wells per condition were examined in three independent experiments. To explore the involvement and role of QE-NCs, cells treated with DMEM alone were used as a negative control.

### 4.5. Cell Adhesion Assay

The effect of QE-NCs on the adhesive properties of A459 cells was determined by the adhesion assay. Twenty-four-well plates (BD Biosciences, San Jose, CA, USA) were blocked with 10 µg/mL IV collagen in DMEM. After cell adhesion was achieved, the culture medium was replaced with 0, 15, or 30 μmol/L QE-NCs of three different sizes or 3 μg/mL cisplatin. The cells were incubated for another 24 h. Cells were seeded at 1 × 10^5^ cells/well with serum-free medium in the coated 24-well plates. After incubation for 30 min, the cells were washed with PBS, fixed with 4% paraformaldehyde, stained with 0.1% methyl violet for 10 min, permeabilized with 0.2% Triton X-100, and measured spectrophotometrically at 550 nm. The adhesion rate was calculated using the following formula: Cell adhesion rate (%) = As/Ac × 100%. The experiment was repeated six times.

### 4.6. Wound Healing Assay

Wound healing assay was employed to assess cell migration. Cells were seeded in 6-well plates and cultured until they reached confluence. A wound was created by manually scraping the cell monolayer with a sterile 20 μL pipette tip. Cells were washed twice with PBS to remove the floating cells and then incubated in DMEM supplemented with 3% FBS. Then, QE-NCs with different particle sizes at 0, 15, or 30 μmol/L or 3 μg/mL cisplatin was added. Cell migration was observed at pre-selected time points (0 and 24 h). Images were acquired using a Nikon DS-5M Camera System mounted on a phase-contrast Leitz microscope (100×). The migration rate was calculated using the following formula: Cell migration rate (%) = Ss/Sc × 100%, where Ss is the experimental area and Sc is the control area. The experiment was repeated three times.

### 4.7. Matrigel Invasion Assay

Cell invasion abilities were explored using transwell chambers. The upper chamber of the transwell was pre-coated with Matrigel 1:9 (*v*/*v*) overnight at 37 °C. The various formulations mentioned in the migration assay were added to A549 cells, and cells were incubated for 24 h at 37 °C in 5% CO_2_. After digestion of the pre-cultured A549 cells and the control cells, cell suspensions were prepared in pure DMEM at 5 × 10^5^/mL. To each chamber, 200 μL of cell suspension was added to obtain approximately 1 × 10^5^ cells in each well. DMEM containing 10% FBS was added to the lower chamber to stimulate cell migration. Following incubation at 37 °C in 5% CO_2_ for another 24 h, non-migrated cells on the upper surface were gently removed by a cotton swab. Cells that migrated to the lower compartment of the chamber were fixed in 4% paraformaldehyde and stained with 0.1% methyl violet for 10 min. Then the fixed and stained cells were eluted with 33% acetic acid solution, and the eluent measured spectrophotometrically at 570 nm. At least three chambers were counted for each experiment.

### 4.8. Western Blot Analysis

The A549 cells were seeded at a density of 1 × 10^6^ cells/dish in 10 cm cell culture dishes. After cell adhesion was achieved, the cells were divided randomly into three groups: the negative control group was cultured with DMEM alone; 3 μg/mL cisplatin was used as the positive control group; the culture medium was replaced with 0, or 30 μmol/L QE-NCs with three different particle sizes in the experimental groups. The concentration of 15 μmol/L was added to 200 nm particle size groups, investigating the effect of concentration. After culturing for another 24 h, cells were collected and lysed with lysis buffer. Cell lysates were centrifuged (10 min at 10,000× *g*, 4 °C) and the supernatants were measured using a BCA protein quantitation assay. Next, 30 μg of protein was separated with 10% sodium dodecyl sulfate polyacrylamide gel electrophoresis (SDS-PAGE), transferred to 0.22 μm PVDF membranes, and blocked with 5% bovine serum albumin (BSA) in PBST. Each membrane was incubated with a specific primary antibody STAT3 (1:800) and β-actin at 4 °C overnight. Membranes were washed with PBST and incubated with the appropriate secondary antibody (1:1000) for 2 h at room temperature. Visualization was carried out with the ECL Western blotting detection kit according to the recommendations for use. Pictures were analyzed using ImageJ 1.46r analysis software. Each sample had three parallel wells.

### 4.9. Statistical Analysis

All quantitative results are presented as the mean ± standard deviation. Statistical analysis was performed with PRISM 5.0 (GraphPad Prism, San Diego, CA, USA). Comparisons between control and treated groups were conducted using one-way analysis of variance (ANOVA) with Student’s t-test (two-tailed). *p* < 0.05 was considered statistically significant.

## 5. Conclusions

In this study, QE-NCs were successfully developed to deal with the drawbacks of QE, and the biological effects of QE-NCs of different particle sizes were systematically presented from the cellular to the molecular level. It was obvious that QE-NCs had a negative impact on the proliferation, migration, and invasion of A549 cells. The particle size of QE-NCs had a remarkable influence on biological efficacy, such that QE-NCs with a smaller particle size improved the anti-tumor effect of QE. In conclusion, NCs provide a potentially favorable and feasible choice for QE in anti-cancer research, and it is critical to optimize the particle size of QE-NCs to make them appropriate and stable for therapeutic purposes.

## Figures and Tables

**Table 1:** Preparation process and characterization of the three quercetin nanocrystals (QE-NCs) with different particle sizes (*n* = 3).

Number	Milling Speed (rpm)	Milling Time (min)	Size (nm)	PdI	Zeta Potential (mV)
1	3000	4	217.28 ± 6.24	0.21 ± 0.09	−28.43 ± 0.85
2	2500	2	501.51 ± 58.35	0.25 ± 0.02	−21.27 ± 0.59
3	1500	4	3146.33 ± 105.14	0.29 ± 0.06	−19.43 ± 1.35

**Table 2:** The IC_50_ values of QE-NCs of different particle sizes on A549 cells (*n* = 6).

Sizes	IC_50_ (μmol/L)		
	24 h	48 h	72 h
200 nm	47.03 ± 16.64	37.88 ± 6.46	29.99 ± 6.14
500 nm	62.25 ± 15.65	48.79 ± 7.95	28.59 ± 8.04
3 μm	77.06 ± 12.29	50.82 ± 11.32	28.92 ± 6.76

**Table 3:** The adhesion rate of QE-NCs of different particle sizes on A549 cells (*n* = 6).

Concentrations	Adhesion Rate (%)			
	200 nm	500 nm	3 μm	Cisplatin
15 μmol/L	56.60 ± 16.44 *	68.53 ± 17.81 *	74.87 ± 26.43	/
30 μmol/L	50.76 ± 12.43 *	58.79 ± 9.39 *	63.58 ± 10.11	/
3 μg/mL	/	/	/	33.99 ± 7.31 **

* *p* < 0.05, ** *p* < 0.01, compared with the blank control group.

**Table 4:** The mobility of QE-NCs of different particle sizes on A549 cells (*n* = 3).

Concentrations	Migration Rate (%)			
	200 nm	500 nm	3 μm	Cisplatin
15 μmol/L	33.00 ± 5.39 **^,^^##^	50.56 ± 2.70 **^,^^##^	68.81 ±4.03 *	/
30 μmol/L	19.48 ± 3.74 **^,^^##^	42.74 ± 8.08 **	50.51 ± 6.75 **	/
3 μg/mL	/	/	/	15.32 ± 5.30 **

* *p* < 0.05, ** *p* < 0.01, compared with the blank control group. ^##^ *p* < 0.01, compared with the 3 μm particle group.

**Table 5:** The invasion of QE-NCs of different particle sizes on A549 cells (*n* = 3).

Concentrations	Invasion Rate (%)			
	200 nm	500 nm	3 μm	Cisplatin
15 μmol/L	57.17 ± 1.02 **^,^^##^	58.54 ± 9.28 **^,^^#^	81.91 ± 3.08 **	/
30 μmol/L	39.97 ± 5.34 **^,^^##^	41.32 ± 4.34 **^,^^##^	70.97 ± 7.29 **	/
3 μg/mL	/	/	/	27.09 ± 1.01 **

** *p* < 0.01, compared with the blank control group. ^#^ *p* < 0.05, ^##^ *p* < 0.01, compared with the 3 μm particle group.
